# THz quantum gap: exploring potential approaches for generating and detecting non-classical states of THz light

**DOI:** 10.1515/nanoph-2023-0757

**Published:** 2024-01-29

**Authors:** Yanko Todorov, Sukhdeep Dhillon, Juliette Mangeney

**Affiliations:** Laboratoire de Physique de l’Ecole normale supérieure, ENS, Université PSL, CNRS, Sorbonne Université, Université Paris-Diderot, Sorbonne Paris Cité, Paris, France

**Keywords:** terahertz, quantum technology, light–matter interaction, nanostructures

## Abstract

Over the past few decades, THz technology has made considerable progress, evidenced by the performance of current THz sources and detectors, as well as the emergence of several THz applications. However, in the realm of quantum technologies, the THz spectral domain is still in its infancy, unlike neighboring spectral domains that have flourished in recent years. Notably, in the microwave domain, superconducting qubits currently serve as the core of quantum computers, while quantum cryptography protocols have been successfully demonstrated in the visible and telecommunications domains through satellite links. The THz domain has lagged behind in these impressive advancements. Today, the current gap in the THz domain clearly concerns quantum technologies. Nonetheless, the emergence of quantum technologies operating at THz frequencies will potentially have a significant impact. Indeed, THz radiation holds significant promise for wireless communications with ultimate security owing to its low sensitivity to atmospheric disturbances. Moreover, it has the potential to raise the operating temperature of solid-state qubits, effectively addressing existing scalability issues. In addition, THz radiation can manipulate the quantum states of molecules, which are recognized as new platforms for quantum computation and simulation with long range interactions. Finally, its ability to penetrate generally opaque materials or its resistance to Rayleigh scattering are very appealing features for quantum sensing. In this perspective, we will discuss potential approaches that offer exciting prospects for generating and detecting non-classical states of THz light, thereby opening doors to significant breakthroughs in THz quantum technologies.

## 1 Introduction

The emerging realm of quantum technologies promises unprecedented advances in fields such as sensing, high-performance computing, simulation, cryptography, and metrology. These technologies have been so far implemented predominantly in the microwave and optical regimes and have an untapped potential in the terahertz (THz) spectral range. Exploiting this frequency domain could have a number of benefits. For instance, quantum cryptography at THz frequencies is of considerable interest for wireless communications with ultimate security, as THz wireless links exhibit attenuation levels several orders of magnitude lower than free-space optical links in the presence of dust, fog, and atmospheric turbulences [1]. In addition, extending the transition frequency of most solid-state qubits (superconducting circuits or semiconductor spins) from the GHz to the THz spectral range will support efforts on increasing the operating temperature of qubits to use a simple ^4^He pumping system and thus overcome the scaling problems posed by limited cooling power of dilution refrigerators below 100 mK. This will also enable qubits to be manipulated at higher speeds. Furthermore, almost all polar molecules exhibit unique spectral signatures in the THz frequency range arising from transitions between rotational quantum levels [2]. As a result, THz radiation can manipulate the quantum states of many molecules, which are recognized as new platforms for quantum computation and simulation with long range interactions [3]. Finally, the ability of THz radiation to penetrate materials that are typically opaque, or its resistance to Rayleigh scattering are very valuable features for quantum applications such as quantum telecommunications and quantum sensing [4].

Non-classical states of light, such as single photons and squeezed light, are fundamental building blocks of quantum technologies with applications in quantum communication, quantum computation, quantum simulation, quantum sensing [5], [6], and quantum metrology. In this perspective, we will discuss some potential approaches that hold promise for generating and detecting non-classical states of THz light, thereby paving the way for ground breaking advancements in THz quantum technologies.

## 2 Generating non-classical states of THz light

In principle, any nonlinear process can produce quantum light [7], [8]. Non-linear optical processes in solid-state materials are the most widely used approach to generating quantum light. Significant progress in the generation of quantum light, including single photons, entangled photon pairs and quadrature-squeezed states, has been made over the last few decades in the fields of optics [9]–[11] and microwaves [12]–[14]. However, in the THz spectral range, the generation of squeezed light has remained elusive until now. In this section, we will present promising approaches, exploiting recent advances in nanostructures, THz components and devices, to produce THz squeezed light.

### 2.1 THz quantum cascade lasers (QCL)

**Figure 1:**
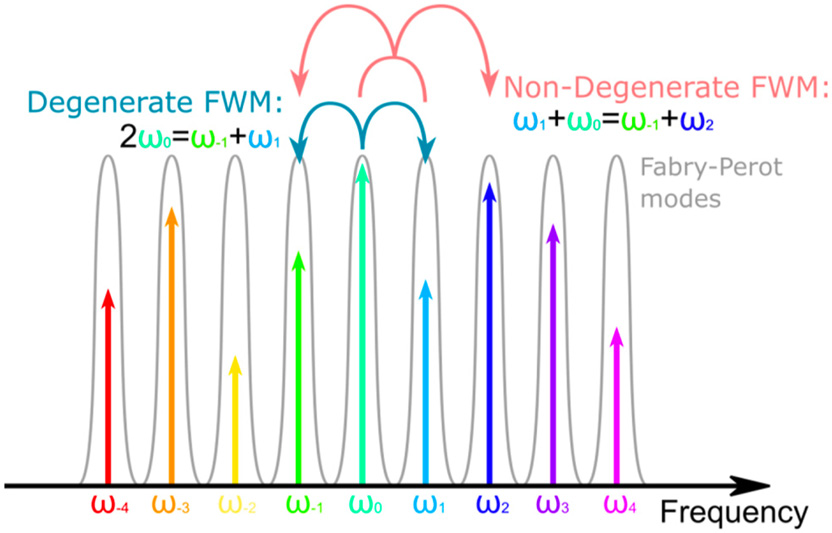
Schematic representation of frequency comb formation mechanisms through degenerate and nondegenerate four-wave mixing (FWM). Ref. [20] licensed under Creative Commons Attribution 4.0 License.

THz QCLs are, to date, the most efficient miniaturized lasers at THz frequencies with impressive improvements in performance over the last few years, including Peltier temperature operation with milliwatt output powers [15]. Their broad gain and controlled group velocity dispersion has recently enabled compact frequency comb (FC) generation, based on four-wave mixing (FWM) processes that take place within the gain medium [16], [17]. This third order nonlinear process permits the generation of quantum correlated spectral modes where, in the simplest form, a strong central mode (*ω*_*p*_) permits the generation of two correlated sidebands, the signal (*ω*_*s*_) and idler (*ω*_*i*_), where 2*ω*_*p*_ = *ω*_*s*_ + *ω*_*I*_ (see Figure 1). As a consequence, QCLs are ideal candidates for the generation of multi-mode squeezed states of light. This permits perspectives towards THz quantum photonic platform based on chip scale quantum emitters, enabling the generation of non-classical THz radiation and/or demonstration of entanglement among different comb-emitted modes, in close analogy with squeezing effects demonstrated 25 years ago in semiconductor bipolar laser diodes [18], [19]. They can then be potentially configured as the founding blocks, in the THz spectral range, for the development of the ultimate sensitivity in spectroscopic and sensing measurements, or to increase capacity, robustness and security of selected free-space quantum communication channels.

### 2.2 A two-level system strongly coupled to a THz cavity

**Figure 2:**
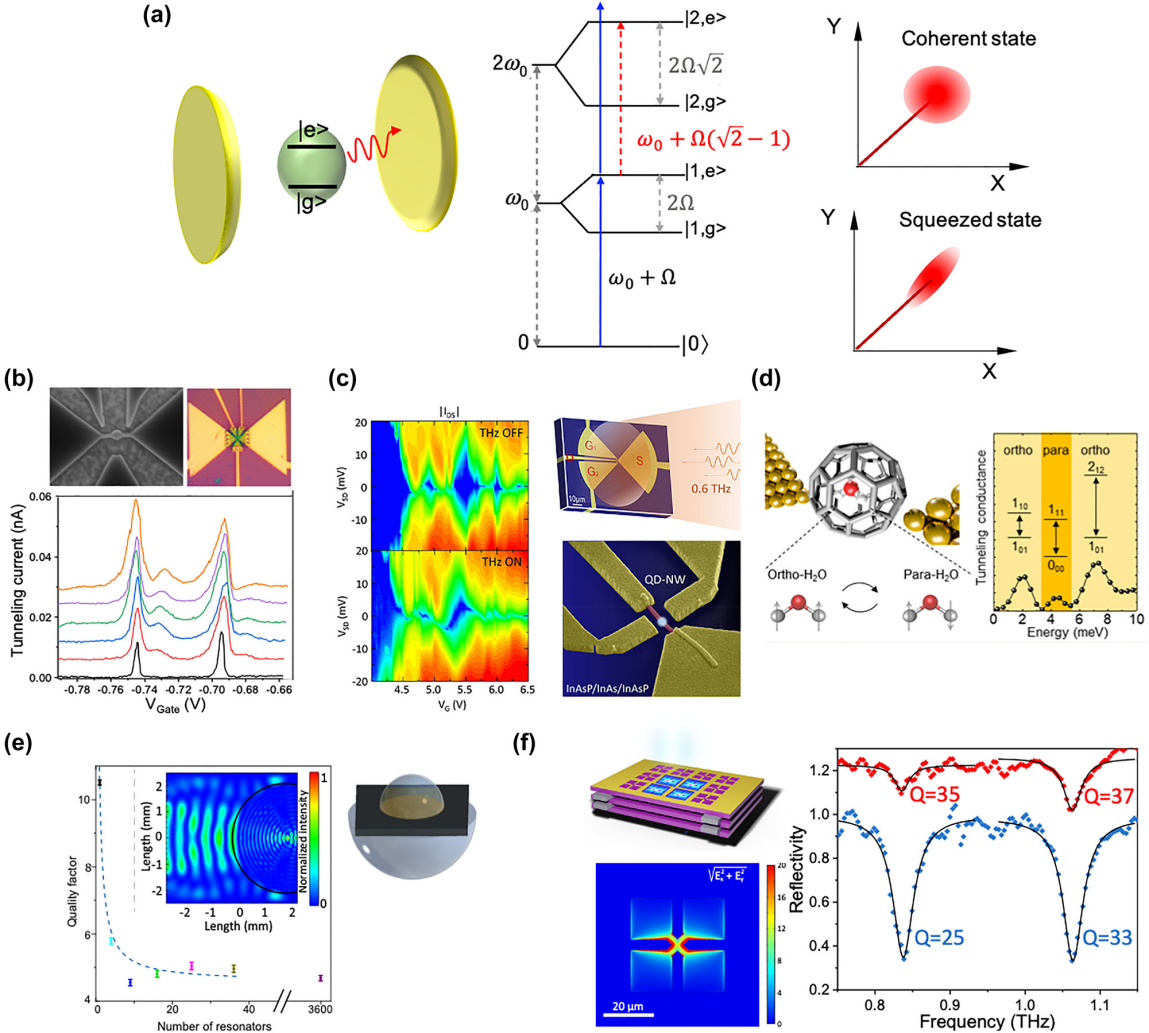
THz two-level systems and resonators. (a) A two-level system in cavity (left). Anharmonic spacing of the levels causes phenomena such as photon blockade and photon-induced tunneling (middle). Coherent and squeezed states of light (right). (b) Images of a single graphene quantum dot transistor. Source-drain current versus gate voltage without (black curve) and with THz irradiation at frequencies from 0.27 THz to 0.38 THz (color curves) revealing satellite peaks due to THz photon-assisted tunneling process. Ref. [22] licensed under Creative Commons Attribution 4.0 License. (c) Low-temperature electrical transport through a quantum-dot nanowire single-electron transistor in the dark and illuminated states (right). Images of a planar on-chip split bow-tie antenna and of the prototypical single-electron transistor (left). Ref. [23] licensed under CC-BY-NC-ND 4.0. (d) THz photocurrent spectroscopy on H_2_O@C_6_0 single molecule transistors, the excitations observed below 10 meV are identified to be the quantum rotational excitations of the water molecule. Reprinted (adapted) with permission from Ref. [24]. Copyright 2021 American Chemical Society [25]. (e) *Q*-factor versus the number of complementary resonating metasurfaces (right). Schematic of the THz resonator and simulation result of the focused THz beam at the interface between the front lens and the resonator field (right). Ref. [26] licensed under Creative Commons Attribution 4.0 License. (f) Representation of a Tamm cavity/LC metamaterial coupled LC resonator structure. Electric field enhancement factor in the LC metamaterial plane over a single unit cell (left). Reflection spectra of a Tamm cavity resonant at approximately 0.95 THz coupled with an LC metamaterial resonant at 0.92 THz (right), from Ref. [27]. 2023 Optica Publishing Group.

Alternative approaches to generating non-classical states of light are based on strong coupling between microcavity photons and quantum emitters, which in this specific case are required to be in the form of a two-level system. The strong coupling regime, achieved when the interaction between the emitter and the cavity mode surpasses the cavity loss and emitter decay, induces large optical nonlinearity at the single photon level that can be harnessed to produce non-classical light exhibiting squeezing properties [21]. As shown in Figure 2, the energy eigenstates of a two-level system strongly coupled on resonance to an optical resonator are grouped into manifolds of two-level dressed states with a non-constant energy difference between consecutive manifolds, leading to anharmonic spacing. A coherent probe beam resonant with the first-order manifold is detuned from transitions to the second manifold. Thus, once a photon is coupled to the system, it suppresses the probability of coupling a second photon of the same frequency, resulting in photon blocking. Similarly, the absorption of a first photon tuned close to the bare cavity resonance enhances the absorption of subsequent photons due to resonance with higher-order manifolds, leading to photon-induced tunneling. As a result of these two effects, the output field acquires sub-Poissonian statistics. The squeezed light stems from the quantum coherence of photon pairs emitted from the system.

Squeezed light generation based on strong coupling of a two-level system to a cavity has previously been demonstrated in the optical range, for example in an InAs quantum dot coupled to a photonic crystal resonator [28], or in a single atom of Rb placed in a high finesse optical resonator [29]. This technique has also been also implemented in the microwave range, for example using a two-level system based on a Cooper pair box strongly coupled to a microwave coplanar resonator [30]. However, this approach remains elusive in the THz spectral range so far. In the perspective of generating squeezed light in the THz domain, the transition energy of the two-level system must be tuned in the range of few meV and the quality factor *Q* of the THz resonator must be high.

Two-level systems with THz resonance frequencies are mainly achieved in semiconductor quantum dots, molecules, impurity, and Rydberg atoms (see Figure 2). For instance, graphene quantum dots (QD) [31] of few tens of nanometers in diameter, defined by physical etching [22], [32], display few meV energy level spacings. These graphene QDs are very promising for THz quantum optics because they exhibit an ultrasensitive response to THz photons [33], [34] and a large THz electric dipole (*d* – 230 nm) [23]. Currently designed with a transition frequency in the tens of GHz, coupled double QDs obtained by electrostatic confinement in bilayer graphene open interesting perspectives as a two-level system in the THz spectral [35], as this approach prevents from localized states at the graphene edges. Gate defined QDs with a THz frequency transition have been also recently achieved using a carbon nanotube [36], a InAs/InAs_0.3_P_0.7_ quantum-dot nanowire [37] and a AlGaAs/GaAs two-dimensional electron system [25]. Alternatively, colloidal HgTe QDs of typical diameters ∼100 nm, produced by a bottom-up technique, also possess energy level spacing of few meV [24]. Several molecules are also promising as a two-level system in the THz spectral range as their vibrational and rotational modes resonate at THz frequencies. For example, individual molecule of H_2_ has been revealed as a two-level system with its coherent superposition exhibiting extreme sensitivity to THz electric field [38]. As well, low-energy vibrational modes of a single C_60_ molecule [39] and quantum rotational excitations of a water molecule have been observed in the few meV range [40]. In addition, coherent two- and three-state superpositions of the phosphorus impurity (donor) with hydrogenic states in silicon have been prepared using THz radiation [41]. At last, Rydberg atoms in a thermal vapor are characterized by strong response to electromagnetic fields with THz transitions between Rydberg levels [42], [43].

THz resonators with a high-quality factor, *Q* > 200, are mostly Fabry–Perot cavities [26], [27], [44], [45]. Their mode volumes are very large because they are limited by diffraction, *V* > (*λ*/2)^3^, which leads to very poor overlap with any sub-micrometer two-level system, such as QDs. To overcome this issue, hybrid resonators based on a Fabry–Perot cavity couple to an electronic circuit such as an LC resonator have recently been developed (see Figure 2). Indeed, LC resonators show low *Q* (∼10) but provide subwavelength mode volume, *V* < 10^−5^ *λ* ^3^ [46]. Hybrid resonators have recently made it possible to achieve both high *Q* and low *V* [47], [48]. The optimization of the light–matter interaction with nanoscale quantum objects is also a very relevant topic for quantum detectors of THz photons (see further).

In summary, the building blocks to achieve microcavity coupled two-level THz emitters are well present today. The next step will be to demonstrate THz squeezed radiation states from such systems, which would allow envisioning a road for performing quantum optics experiments in the THz frequency domain. However, significant effort should be provided also on the detector side, as discussed in the next section.

### 2.3 Ultra-strong coupling (USC) regime of THz light–matter interaction

A very intriguing way of generating non-classical light is the dynamical Casimir effect [49]. This effect consists of the generation of photons from the vacuum state in time modulated systems. It was originally described theoretically for a Fabry–Perot cavity, where one of the mirrors is semi-transparent and moving periodically: the periodic modulation of the vacuum creates radiation [50]. This case is the dynamical analogue of the famous Casimir effect, where the vacuum fluctuations create a net force on the mirrors [51]. Another vision for this effect is the Unruh radiation: this occurs from accelerated bodies because the vacuum in an accelerated frame is actually a thermal state with a finite temperature [52]. Very recently, it was theoretically demonstrated that the detection of Unruh radiation generated for very brief acceleration periods is equivalent to the electrooptic sampling of the vacuum [53], linking this physics to recent experiments in the THz spectral range [54], [55].

**Figure 3:**
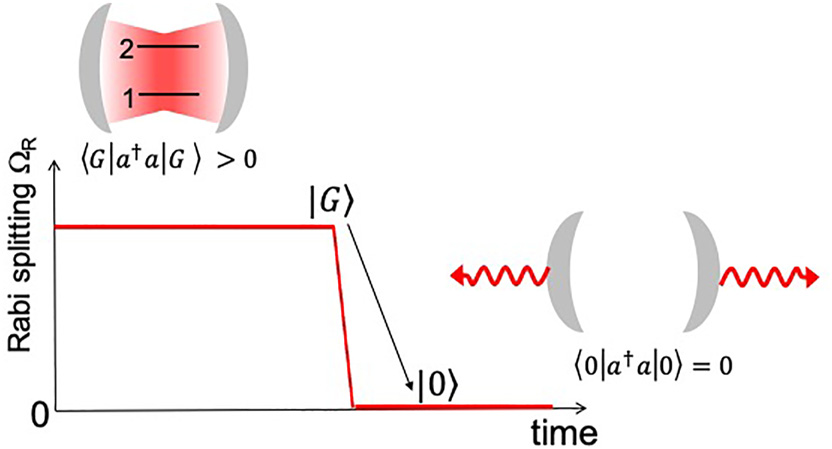
An illustration of the dynamical Casimir effect. The ground state of the USC system is not empty but contains virtual cavity photons. An abrupt turning off of the interaction strength Ω_*R*_ brings the system into an uncoupled case where the ground state of the cavity is an ordinary vacuum without photons. The virtual photons in the ground state are then radiated away in a form of correlated pair of real photons [56].

A first experimental demonstration of the dynamical Casimir effect was published in 2011 from a group of the University of Chalmers [51], who achieved a fast modulation of the modes of a transmission line resonator terminated by an SQUID. In 2005, Ciuti et al. [56] proposed theoretically a radically different approach, based on the electronic transitions in a semiconductor quantum well strongly coupled to a microcavity mode. Namely, Ciuti et al. examined the case where the light–matter coupling strength, Ω_*R*_, becomes a sizable fraction of the transition frequency *ω*_21_. One thus obtains a novel regime of light–matter interaction, dubbed ultra-strong coupling (USC) regime. This regime is characterized by the breakdown of the rotating wave approximation, usually employed to solve the quantum dynamics of the system. As illustrated in Figure 3, in this regime the ground state of the coupled system, $\left|G\right\rangle$ , can no longer be written as a tensor product between the ground states of the two uncoupled systems, $\left|0\right\rangle ⊗\left|F\right\rangle$ . As a result, the ground state of the USC system acquires a non-zero population of cavity photons $\left\langle G\right|a^{†}a\left|G\right\rangle ∼{\left(\Omega_{R}/\omega_{12}\right)}^{2}$ . Now, if the coupling constant Ω_*R*_ is switched back non-adiabatically to zero, the vacuum state does have a zero-photon number $\left\langle 0\right|a^{†}a\left|0\right\rangle =0$ , and the excess of virtual photons present in $\left|G\right\rangle$ are released in a form of correlated pair of real photons. The intensity of this Casimir radiation depends on the rate on which Ω_*R*_ is modulated [57]. The excited states of the system, the cavity polaritons, also acquire non-trivial properties and become squeezed states [51], [58], [59]. The adequate description of the emission from such states requires careful theoretical analysis [60].

Currently, there are many implementations of the USC regime, across a vast frequency range of the electromagnetic spectrum [61]. Specifically in the THz range USC has been achieved with the electronic transitions in highly doped quantum wells [62], as well as metamaterial-coupled cyclotron resonances [63]. Yet, experimental evidence of the intriguing phenomena of dynamical Casimir effect, which initiated the story of the USC is still missing. In the THz frequency range, a very promising approach is the ultra-fast time modulation of the Rabi constant Ω_*R*_ through optical pumping, which has been shown both in the case of intersubband polaritons [64], as well as Landau level polaritons [65]. The real obstacle is the detector sensitivity, as the Casimir radiation is intrinsically very week. Thus, a real effort should be provided in building very sensitive THz detectors, which is the object of the next section. However, it is clear from the above example that the THz domain provides both the devices and tools for the experimental observations of intriguing quantum electrodynamical phenomena that would be difficult to observe in other spectral domains. Another appealing approach would be to study USC in the case of few electron systems [34], [66], [67]. In that case, both the features of the USC and the intrinsic quantum fermionic non-linearities would contribute for generating non-classical states of light: still an uncharted territory both for theoreticians and experimentalists.

## 3 Detecting non-classical states of THz light

Because of the ultralow photon energies at THz frequencies, photodetectors in this spectral range are notoriously underdeveloped and broadband detectors of single photon or non-classical states of light are nonexistent*.* Only one demonstration of a single THz photon detection has been reported thus far, in a narrow band around 1.5 THz, using a quantum capacitance detector [68]. In this superconducting device, which has a noise-equivalent power of less than 10^−20^ WHz^−1/2^, the free electrons produced by photons in a superconductor penetrate a small capacitive island integrated in a resonant circuit. Besides, recent theoretical studies have predicted that superconducting magic-angle bilayer graphene device is capable of detecting single photons of ultralow energies (i.e. at THz frequencies) by utilizing its record-low heat capacity and sharp superconducting transition [69]. Here, we present alternative promising approaches to detect single THz photon with ability for photon number resolution.

Of particular interest are single photon detectors that can be realized with semiconductor nanostructures. Such realizations have been pioneered from Prof. S. Komiyama’s group from University of Tokyo [70]. The main idea of the device is to build separate absorbing region in a form of semiconductor island, separated by strong and controllable potential barriers from the rest of the device, thus resulting in a single-electron transistor. After photon absorption, the photoexcited electron is transferred in a nearby conducting channel, where it is accelerated towards a read-out circuit. The key advantage of that device is that the photon absorption changes the charging state of the insulated island, which can be read-out with a high precision [66]. Furthermore, the charge excitation has typically very long lifetime: milliseconds and even seconds [27]. Sensing a charge difference that corresponds to a single electron is then equivalent to the readout of an event that corresponds to the absorption of a single photon.

**Figure 4:**
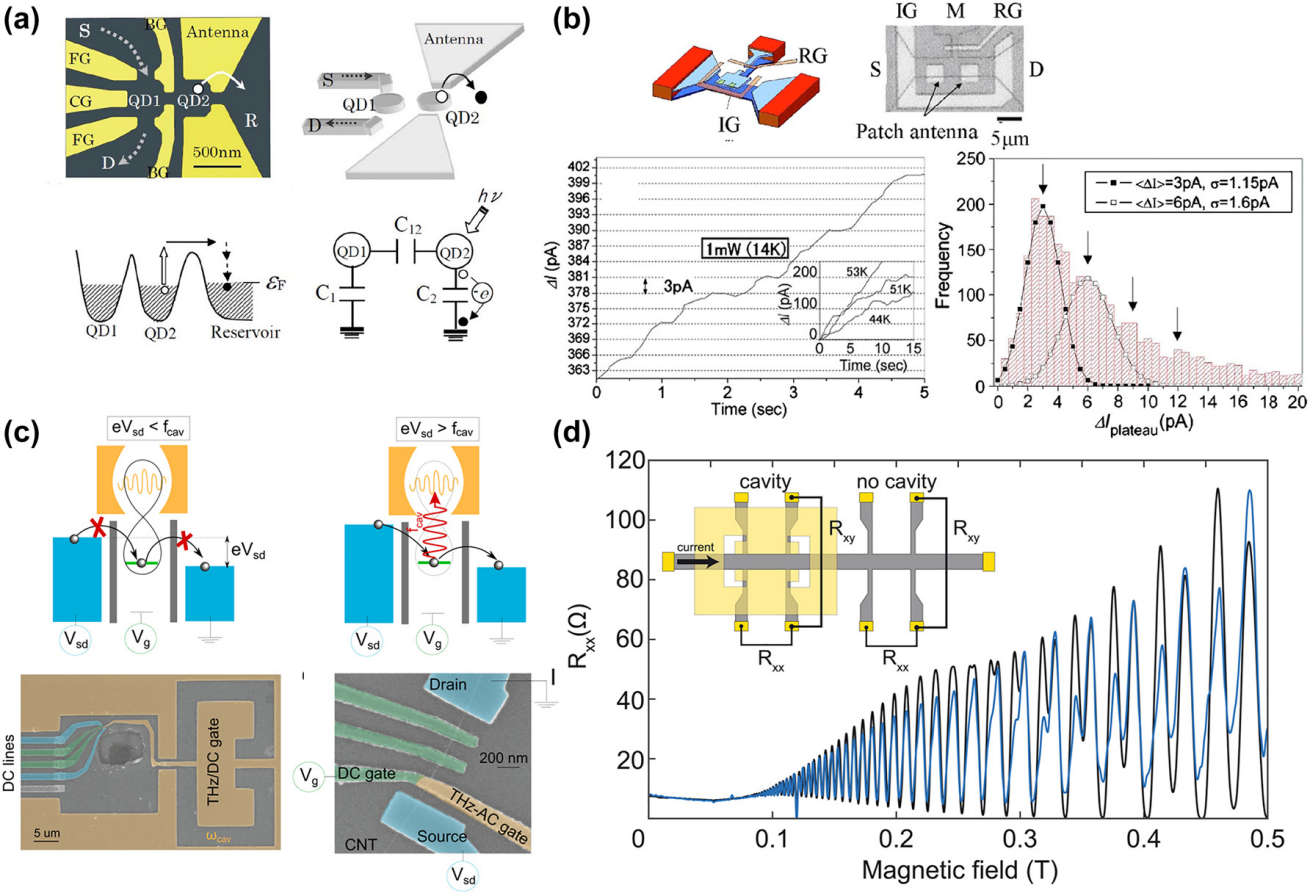
Detection of non-classical states of light. (a) Working principle of a single-photon detector in the microwave range formed by two capacitively coupled GaAs/Al_*x*_Ga_1−*x*_AsGaAs/AlxGa_1−*x*_As parallel quantum dots. Reproduced with permission from Ref. [68], Copyright 2002, American Chemical Society. (b) Schematic representation and a microscope image of a charge-sensitive infrared phototransistors (up). Time trace of the photocurrent at a temperature of 14 K. The histogram of the frequency of stepwise. Reproduced with permission from Ref. [74], Copyright 2002, American Chemical Society. (c) Cartoon pictures of the process leading to a THz transport gap in a carbon nanotube quantum dot (up). SEM picture of the devices: the THz cavity is capacitively coupled to the QD in the carbon nanotube (bottom). Ref. [34] licensed under Creative Commons Attribution 4.0 License. (d) Resistance for a reference Hall bar (black lines) and for a complementary THz split-ring 140 GHz resonator embedding a Hall bar revealing a breakdown of the topological protection by cavity vacuum fields in the integer quantum Hall effect. Reproduced with permission from Ref. [76], reprinted with permission from AAAS.

A first implementation of that idea in the THz range (∼1.7 THz) was realized with a single quantum dot under strong magnetic field [71]. The quantum dot was defined by gating 2D electron gas in GaAa/AlGaAs heterostructure. A double-dot device without magnetic field and operating at 500 GHz was also demonstrated [72]. In both cases, impressive NEPs on the order of 10^−21^ W/Hz^0.5^ were reported by the authors. In the microwave, that approach has been the subject of several theoretical studies. Modeling predicts that a double quantum dots nearby a charge detector, coupled to a high-*Q* microwave cavity, enable single photon detection and furthermore photon counting [73], [74] (see Figure 4). Indeed, for ideal, unity efficiency detection, the fluctuations of the charge current reproduce the statistics of the incoming photons [75].

Another implementation was achieved in the mid-infrared range, in the wavelength range *λ* = 9 µm–15 µm. In that case the detector design makes use of the same type of band-structure engineering that is used for unipolar devices [77]. The absorbing island is obtained from a thin quantum well that is connected with a conducting source-drain channel trough a triangular barrier [78]. The device, called charge sensitive infrared phototransistor (CSIP) is processed in a transistor architecture with several gates, which allow lateral insulation of the thin well as well as a reset action. Typically, the device is operated in a non-stationary regime, where the source-drain current increases slowly with time owe to the transfer of photoexcited electrons. This is the regime of photon counting. For devices where the absorbing area is small enough (∼10 µm^2^), the variations of the source-drain current are step-like, where each step corresponds to the detection of a photon [79]. Statistics of the size of the current steps can be related to the statistics of the photon source. In Ref. [74] this was demonstrated with a thermal source, where the authors observed a Poissonian distribution of the photon arrival events. Clearly, it is very appealing to apply the same type of detector scheme to quantum sources, where one wants to observe the quantum fluctuations of the source. Another very interesting perspective is to combine several CSIP devices in order to perform time-delayed correlation measurements of the source. However, in order to reach ultimate sensitivity of CSIPs it is imperative to reduce as much as possible its absorbing area, such as the absorption of a single photon roughly corresponds to the read-out of a single photoexcited electron [74]. To achieve this ambitious task, an interesting perspective is to employ advanced photonic architectures such as antenna-coupled metamaterials [80]–[83]. The THz range is particularly suitable for such studies, as relatively low metal loss, long wavelengths and advanced nanofabrication techniques allow exploring complex geometries for optimized subwavelength light confinement [27], [78], [84]. It should also be noted that such strategies can also lead to an improved quantum efficiency of the detectors, which has been reported to be rather low 0.1 %–1 % [67], [68], [74]. The low quantum efficiency is nevertheless not always an issue in quantum optical experiments, as the second order correlation function g2(0) is independent from it [76].

Another line of research is exploring devices operating in the ultra-strong light matter coupling regime (see Figure 4(c) and (d)). In the MIR range, this research work has been going on for at least two decades, and has been concerning mainly unipolar detector devices [85]–[87]. The latter are formidable platform for exploring the interplay between electronic transport and light–matter interaction, however still underemployed at THz frequencies. In the THz frequency range, new types of devices have been emerging [34], [35], [88], with the specific task to probe the quantum fluctuations induced by the USC regime [89]. Clearly, we arrive at a point where the THz community can strongly benefit from these developments, which are highly appealing for the realization of practical THz single-photon detectors and, more generally, the detection of non-classical states of THz light.

Furthermore, detecting the multi-mode squeezed states of light potentially generated by QCLs is a challenging task. Despite improvements over the last few years [90], detecting these quantum states requires improvements in quantum efficiency of THz detectors, such as those based around graphene transistors and improving their response through, for example, reducing the channel lengths [54], [55]. An alternative approach would be the use of coherent detection in the temporal domain using sampling techniques. This approach permits the sensitive detection of the time resolved electric field and has been applied to give the direct aspect of vacuum fluctuations in the mid-infrared [91]. Recent work has extended such concepts to show electric field correlations of THz pulses using cooled electro-optic crystals [92]. However, the response of this approach reduces at high THz frequencies (where THz QCLs operate), despite recent important investigations using on-chip electro-optic geometries. Coherent ultrafast THz photoconductive detectors can provide a potential solution here where their performances can be enhanced by engineering their THz and optical response. For example, the photoconductive material can be engineered into a resonant THz metal-insulator-metal cavity [93], permitting the coherent response to be enhanced by a factor 10 at high THz frequencies. Further, this can be combined with plasmonic interdigitated top contacts to simultaneously enhance the collection efficiency of the photoexcited carriers and the photon absorption [94]. On the actual THz QCL side, considerable efforts are being made in realising harmonic comb operation [95] to produce a spectrum of equidistant modes separated in frequency by a multiple (two to tens) of the natural Fabry–Perot mode spacing defined by the laser cavity. The advantage of a harmonic comb is that it distributes its optical power among few high-power modes, in contrast to the many weaker adjacent cavity modes of a standard dense comb. As well as facilitating the possibility of detecting quantum correlations, this enables the modes of the QCL to be easily separated (using for example, gratings) for correlation measurements.

## 4 Conclusion: the THz quantum gap

For many decades, the THz frequency range was referred as a technological gap in terms of the lack of viable solutions for the generation and detection of THz waves [96]. In the recent years, we have been witnessing impressive developments in terms of both emitters and detectors with commercial applications [97]. Today, the actual THz gap clearly concerns quantum technologies that have been flourishing in the neighboring spectral domains. In the microwave, superconducting qubits are currently at heart of quantum computers developed both by academia as well as high tech startups and companies. In the visible and telecom domain, quantum cryptography protocols have been demonstrated with satellite links. The THz domain, however, has been considerably lagged behind these impressive developments, despite a high potential to bring together the best of the microwave and optical regions. Based on the results shown in the current perspective, we believe that this situation could change in the next decades, as the THz domain shows a plethora of new opportunities for the development of quantum technologies. We now dispose of all building blocks not only to provide quantum technologies in line with those from the other spectral ranges, but also to open new opportunities. Indeed, the THz spectral domain holds the promise of enhanced wireless communication security, raising the operating temperature of solid-state qubits, enabling new quantum computation and simulation platforms through the manipulation of quantum states in molecules, and offering valuable perspectives for quantum sensing applications. Furthermore, the USC regime which has been already demonstrated with THz devices will permit to explore completely new quantum mechanical concepts such as ultrafast quantum gates [98], non-adiabatic electrodynamics [99] and probing fundamental quantum fluctuation [89], [92]. Also, given rapid advances being made in new quantum materials, such as 2D materials [100], and their easy coupling to Si-photonics, there is a realistic prospect of integrated devices for THz quantum optics on-chip, thus providing new platforms for developing THz quantum technologies.
